# Innovative Insights into Ferroptosis in Oligodendrocytes Following Intracerebral Hemorrhage: Implications for Neuroprotection and Therapeutic Strategies

**DOI:** 10.7150/ijbs.100754

**Published:** 2024-08-01

**Authors:** Fangming Liu, Guohua Wang

**Affiliations:** Department of Neurophysiology and Neuropharmacology, Institute of Special Environmental Medicine and Co-innovation Center of Neuroregeneration, Nantong University, 9 Seyuan Road, Nantong, Jiangsu 226019, China.

The recent study by Gu et al., titled “Single-cell and Spatial Transcriptomics Reveals Ferroptosis As The Most Enriched Programmed Cell Death Process In Hemorrhage Stroke-induced Oligodendrocyte-mediated White Matter Injury” 1, offers a groundbreaking analysis of the cellular and molecular dynamics following intracerebral hemorrhage (ICH). Utilizing cutting-edge sequencing techniques, the authors elucidate the roles of different cell types and programmed cell death (PCD) pathways, focusing particularly on ferroptosis in oligodendrocytes.

## Presentation and detailing of sequencing results

The article excels in presenting comprehensive sequencing results, characterized by detailed and diverse datasets. The authors employ single-cell RNA sequencing (scRNA-seq) and spatial transcriptomics to analyze the cerebral hemisphere at various time points post-ICH. This approach provides significant insights into the temporal and spatial dynamics of glial cells, particularly oligodendrocytes and microglia. Such detailed analysis aligns with previous studies that highlight the importance of sequencing techniques in understanding cell dynamics post-injury 2.

## Innovative focus on ferroptosis and LCN2

One of the standout aspects of the study is the innovative use of multiple databases to score PCD processes, leading to the identification of ferroptosis as the primary mechanism affecting oligodendrocytes post-ICH. The study highlights the upregulation of lipocalin-2 (LCN2), a protein typically elevated in neurological diseases, suggesting its potential as a therapeutic target. This aligns with existing literature that underscores the role of ferroptosis in cell death and its potential therapeutic implications 3.

## Microglia and oligodendrocyte interactions

**Figure 1:**
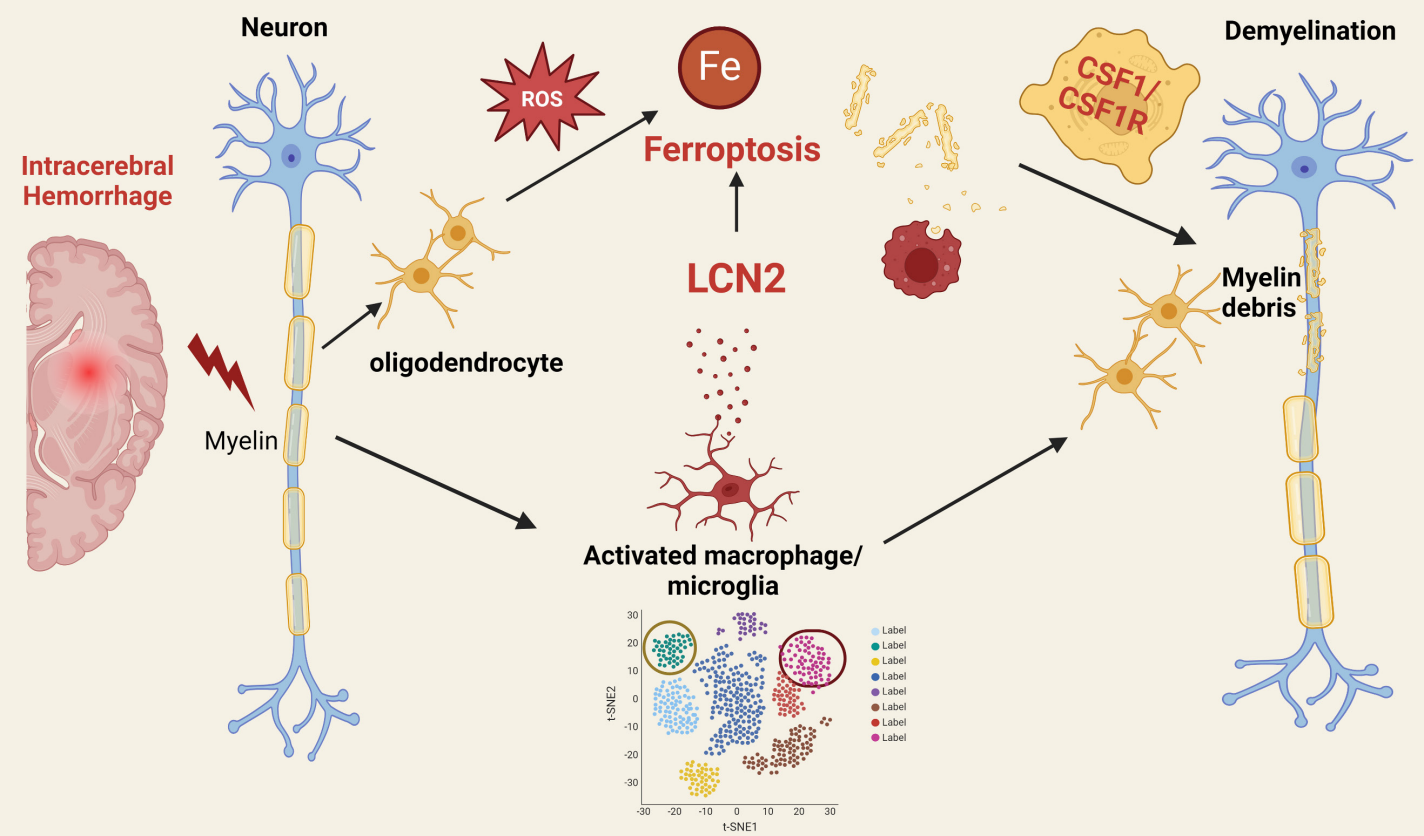
Mechanistic insights into ferroptosis in oligodendrocytes post intracerebral hemorrhage: implications for neuroprotection and therapeutic strategies.

Prior research indicates that activated microglia produce and secrete LCN2, which can have both protective and detrimental effects depending on context and duration 4. Elevated LCN2 levels contribute to initial defense but may also amplify inflammation, resulting in oxidative stress and direct oligodendrocyte damage 4. Chronic microglial activation and sustained LCN2 production contribute to the inflammatory milieu in multiple sclerosis (MS) 5, while in Alzheimer's disease (AD), microglial activation and LCN2 production are linked to neuroinflammation and neuronal damage 6. The authors further explore the interaction between microglia and oligodendrocytes through a co-culture system, which verifies the molecular mechanisms underlying their interplay. The study reveals that microglia positive for LCN2 induce ferroptosis in oligodendrocytes via the CSF1/CSF1R signaling pathway, leading to neurological deficits. This finding provides new directions for neuroprotection research through regulating neuroinflammation and ferroptosis (Figure 1).

## Future research directions

Despite the significant findings, the study opens several avenues for future exploration:

### Experimental validation of ferroptosis mechanisms

Increasing experimental validation of the mechanisms related to ferroptosis is crucial 7. Future studies should employ more experimental methods to detect ferroptosis-related proteins and reactive oxygen species (ROS), enhancing the comprehensiveness and credibility of the findings.

### Sorting and purification of cell subpopulations

Further research should investigate whether the highly expressed subpopulations of oligodendrocytes and microglia identified through sequencing can be sorted and purified. Understanding their specific roles in disease progression could lead to more targeted therapies 8.

### Mechanistic studies of LCN2

The newly discovered protein target, LCN2, warrants further investigation to elucidate its specific mechanisms of action in neurological diseases. Evaluating its potential as a therapeutic target could have significant implications for treatment strategies.

### Expansion of co-culture systems

The co-culture system established in this study provides a valuable platform for investigating neurological diseases. Future studies could apply this system to other neurological conditions to uncover additional interactions and mechanisms 9.

### In-depth molecular mechanisms

Further research should delve into the specific roles of associated molecules, such as CSF1 and CSF1R, in neurological diseases. Exploring their interactions with other molecular pathways could yield new insights into disease mechanisms and potential therapeutic interventions.

## Conclusions

The study by Gu et al. marks a significant advancement in our understanding of the cellular and molecular responses to ICH. By leveraging state-of-the-art sequencing technologies, the authors provide a detailed and layered analysis of the glial cell dynamics and the pivotal role of ferroptosis in oligodendrocyte-mediated white matter injury. Future research building on these findings could pave the way for novel therapeutic strategies targeting ferroptosis and associated pathways in neurological diseases.
